# Molecular Biomarkers of Sessile Serrated Adenoma/Polyps

**DOI:** 10.14309/ctg.0000000000000104

**Published:** 2019-11-26

**Authors:** Priyanka Kanth, Katherine E. Boylan, Mary P. Bronner, Kenneth M. Boucher, Mark W. Hazel, Ruoxin Yao, Stelian Pop, Philip S. Bernard, Don A. Delker

**Affiliations:** 1 Department of Gastroenterology, University of Utah, Salt Lake City, Utah, USA;; 2 Huntsman Cancer Institute, Salt Lake City, Utah, USA;; 3 Department of Pathology, University of Utah, Salt Lake City, Utah, USA;; 4 Division of Epidemiology, University of Utah, Salt Lake City, Utah, USA;; 5 Nemametrix, Salt Lake City, Utah, USA.

## Abstract

**OBJECTIVES:**

Sessile serrated adenoma/polyps (SSA/Ps) contribute up to 30% of all colon cancers. There is considerable histological overlap between SSA/Ps and hyperplastic polyps. Inadequate consensus exists among pathologists, and no molecular biomarkers exist to differentiate these lesions with high accuracy. Lack of reliable diagnosis adversely affects clinical care. We previously defined a novel 7-gene panel by RNA sequencing that differentiates SSA/Ps from hyperplastic polyps. Here, we use the 7-gene panel as a molecular approach to differentiate SSA/Ps and HPs with higher sensitivity and specificity in a large sample set from a tertiary health care center.

**METHODS:**

Reverse transcription quantitative polymerase chain reaction of the 7-gene panel was performed on 223 formalin-fixed, paraffin-embedded serrated polyp and normal colon samples. We compare the sensitivity and specificity of the 7-gene panel with the *BRAF* and *KRAS* mutation incidence in differentiating SSA/Ps and HPs. We also evaluate the clinical data of patients with SSA/Ps showing high and low expression of the gene panel.

**RESULTS:**

The 7-gene RNA expression panel differentiates SSA/Ps and HPs with 89.2% sensitivity and 88.4% specificity. The gene panel outperforms *BRAF* mutation in identification of SSA/Ps. Clinical data suggest that expression of the 7-gene panel correlates with the development of SSA/Ps in the future.

**DISCUSSION:**

This study describes a novel 7-gene panel that identifies SSA/Ps with improved accuracy. Our data show that RNA markers of SSA/Ps advance the distinction of serrated lesions and contribute to the study of the serrated pathway to colon cancer.

## INTRODUCTION

Sessile serrated adenoma/polyps (SSA/Ps) are colon polyps that confer an increased risk for development of colon cancer (1,2) and have been implicated in interval or missed colon cancers (3,4). SSA/P prevalence ranges between 2% and 9% and may be higher than previously thought (5). Current guidelines recommend surveillance colonoscopy for patients with SSA/P(s) in 3–5 years based on polyp size and dysplasia (6). Unlike hyperplastic polyps (HPs), classic SSA/Ps demonstrate unique histological characteristics, including full mucosal thickness crypt dilation and serration, lateral extension of basal crypts, and nuclear dysmaturation. However, many SSA/Ps display these features in only a small number of colon crypts, whereas the remaining polyp has typical HP morphology. Furthermore, most typical HPs show a rare full-thickness dilated crypt or other focal SSA/P features, altogether resulting in significant morphological overlap with HPs. Considerable intraobserver and interobserver variability exists among pathologists in differentiating premalignant SSA/Ps from benign HPs (7–10). Misclassification of serrated lesions can lead to inadequate or unnecessary surveillance colonoscopies, resulting in inappropriate clinical care. Currently, no validated molecular biomarkers are available for accurate detection of SSA/Ps in clinical practice. We recently developed a 7-gene expression panel (*CRYBA2*, *FSCN1*, *MUC6*, *SEMG1*, *TRNP1*, *ZIC2*, *and ZIC5*) that differentiates SSA/Ps from HPs and identifies a subtype of colon cancers likely to develop from SSA/Ps (11).

The serrated colon cancer pathway is an alternate pathway, and CpG island methylation (CIMP high or low), microsatellite instability, and *BRAF* mutation have been shown as possible underlying molecular mechanisms (12–15). Some carcinomas deriving from SSA/Ps may contain *KRAS* mutations, as do the relatively rare traditional serrated adenomas (12,16). Some serrated neoplasia may lack *BRAF* or *KRAS* mutations, and some may not demonstrate microsatellite instability. Hence, not all SSA/Ps or adenocarcinomas arising from the serrated pathway have these changes, and the molecular requirements for progression to cancer remain unclear (16,17). *BRAF* and *KRAS* mutations have been studied in serrated polyps, and SSA/Ps demonstrate higher *BRAF* mutations. However, a large number of HPs also carry *BRAF* mutation, and this solely cannot be used in differentiation of serrated lesions with higher accuracy (18–20).

The goal of this study was to examine our 7-gene panel by RT-qPCR in a larger patient cohort using formalin-fixed, paraffin-embedded (FFPE) tissue. The secondary goal was to compare our 7 gene markers with known genomic markers of SSA/P, which include *BRAF* and *KRAS* mutations in the polyp tissue. Our results further demonstrate gene expression differences between SSA/Ps and HPs and suggest this gene panel could improve and standardize serrated polyp classification.

## MATERIALS AND METHODS

### Patient cohort

Two hundred twenty-three FFPE samples, consisting of 99 SSA/Ps, 78 HPs, 27 uninvolved colon samples from patients with serrated polyps, and 19 control colon samples from patients without colonic polyps on any colonoscopy, were obtained from the University of Utah's pathology tissue core with Institutional Review Board approval. Samples were collected between years 2012 and 2018. Only polyps with less than 10% nonserrated/normal colonic mucosa per block on histologic review were selected to eliminate the need for microdissection. One HP and 3 SSA/Ps were from patients with serrated polyposis syndrome. Clinical data including age, sex, ethnicity, polyp location, and endoscopic polyp size were obtained for all patient samples. Other clinical variables including colonoscopy findings, history of diabetes, aspirin, vitamin D usage, and smoking were obtained as available from the electronic medical record.

### Three-score criteria system

Polyps were reevaluated and scored for 3 criteria by expert gastrointestinal pathologists using anatomical and histological data for each SSA/P and HP sample. A three-score criteria system was used to incorporate all polyp features, including polyp size and location, which may subjectively influence the distinction between SSA/Ps and HPs. The 3 criteria are polyp size >9 mm by endoscopic report, proximal colon location (proximal to the splenic flexure), and predominant classical SSA/P histology (full mucosal thickness crypt dilation and/or serration extending into basal crypts, boot-shaped lateral herniation of basal crypts, and nuclear dysmaturation). The presence of all 3 of these criteria constituted a classic SSA/P status. The presence of 0 criteria constituted a classic HP status.

### RNA and DNA isolations

Ten 10-μm paraffin sections of each FFPE tissue sample were deparaffinized with Neo-Clear (Merck, Darmstadt, Germany), and total RNA was isolated using the High Pure FFPET RNA Isolation Kit (Roche Diagnostics, Mannheim, Germany) or High Pure miRNA Isolation Kit (Roche Diagnostics). Genomic DNA was also obtained from 77 samples using the AllPrep DNA/RNA FFPE Kit (Qiagen, Hilden, Germany) for *BRAF* and *KRAS* mutation detection.

### Reverse transcription quantitative polymerase chain reaction

Two hundred twenty-three colon FFPE samples were analyzed by reverse transcription quantitative polymerase chain reaction (RT-qPCR). Five hundred nanograms of each RNA (measured using a Nanodrop; Thermo Fisher Scientific, Waltham, MA) was reverse transcribed to cDNA using Applied Biosystems High-Capacity RNA-to-cDNA Kit (Product 4387406). The relative mRNA level for each gene was determined using intron-spanning TaqMan gene expression assays (Applied Biosystems, Foster City, CA). Ten-microliter qPCR reactions were performed with Applied Biosystems TaqMan Gene Expression Master Mix (Product 4369016) and 15 ng of cDNA.

### *BRAF* and *KRAS* mutation analysis

Seventy-seven serrated polyp samples with sufficient tissue available for DNA extraction were evaluated for *BRAF* and *KRAS* mutations (0/3 criteria, n = 30; 1/3 criteria, n = 11; 2/3 criteria, n = 11; and 3/3 criteria, n = 25). Primer pairs were designed for PCR amplification and bidirectional Sanger DNA sequencing reactions across *BRAF* codon 600 (V600E) and *KRAS* codons 12–13 (G12D, G12V, G13D).

### Statistical analysis

**Figure 1.**
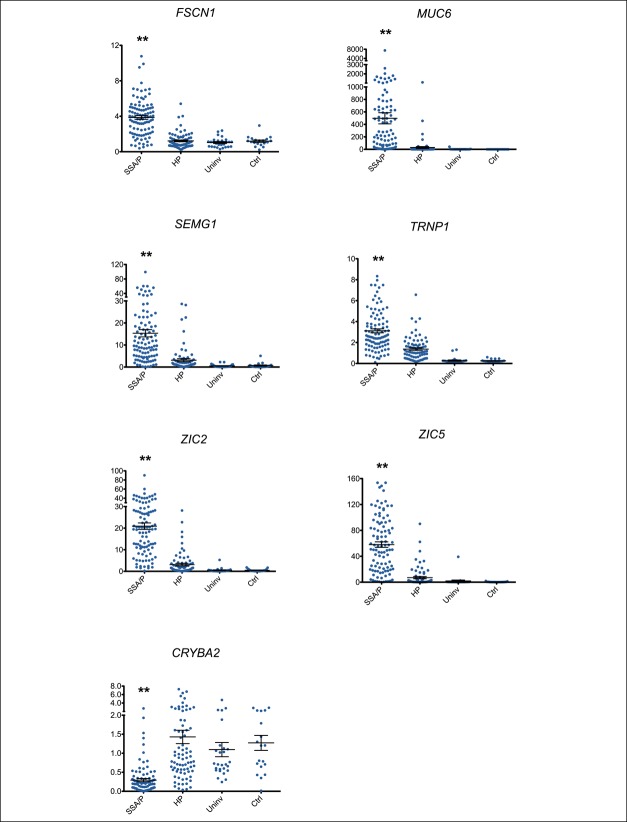
RNA expression by reverse transcription quantitative polymerase chain reaction of the 7-gene panel, graphing each gene individually (*FSCN1*, *MUC6*, *SEMG1*, *TRNP1*, *ZIC2*, *ZIC5*, and *CRYBA2*) in sessile serrated adenomas/polyps (SSA/Ps, n = 99), in hyperplastic polyps (HPs = 78), in uninvolved colon from patients with serrated polyps (Uninvl, n = 27), and in control colon samples from patients without polyps on colonoscopy (Cntl, n = 19). Y axis shows the fold change in expression relative to the mean of HP expression. X axis shows each of the 4 sample groups. Mean ± SE bars are presented for each of the 4 groups. Mann-Whitney *U*-test statistics in GraphPad Prism 5.0d version software (San Diego, CA) were used to determine statistical significance between sample groups. The expression of all 7 genes was statistically significant (**P < 0.0001) between SSA/Ps and the other 3 groups (HPs, uninvolved, and control).

The 7-gene panel was validated using area under the curve (ROC) with a training and test set of samples. The 51-sample training set consisted of polyps fulfilling either none or all of the 3 criteria (SSA/P = 22, HP = 29). Polyps fulfilling the full range of 0, 1, 2, and 3 of 3 criteria were used for the 126-sample test set (criteria 0/3, n = 43; criteria 1/3, n = 18; criteria 2/3, n = 38; and criteria 3/3, n = 27). A sample flow diagram illustrating the different training and test set groups for the study is shown in Figure 1, (see Supplementary Digital Content 1, http://links.lww.com/CTG/A118).

## RESULTS

### Patient cohort and differential expression

We evaluated the expression of our 7-gene panel in 177 serrated polyp (99 SSA/Ps and 78 HPs) and 46 normal colon mucosal (27 uninvolved and 19 control) samples and found significant differential expression (*P* < 0.0001) of each gene between SSA/Ps, HPs, and both groups of normal-appearing colon (Figure 1). Patient demographics for these 223 colon samples are presented in Table 1. As reported in other studies, SSA/Ps were larger and more frequently located in the right colon compared with HPs (6). SSA/Ps were also more frequently observed in female patients although this was not statistically significant (*P* = 0.057). *CRYBA2* was significantly (*P* < 0.0001) underexpressed in SSA/Ps compared with HPs, uninvolved and control colon. *FSCN1*, *MUC6*, *SEMG1*, *TRNP1*, *ZIC2*, and *ZIC5* were significantly (*P* < 0.0001) overexpressed in SSA/Ps compared with HPs, uninvolved and control colon. The fold change for each comparison is shown in Table 1, (see Supplementary Digital Content 2, http://links.lww.com/CTG/A122). These new findings using FFPE tissue RNA are consistent with previous expression studies in our laboratory using RNA from RNAlater-preserved frozen tissues (11).

**Table 1.**
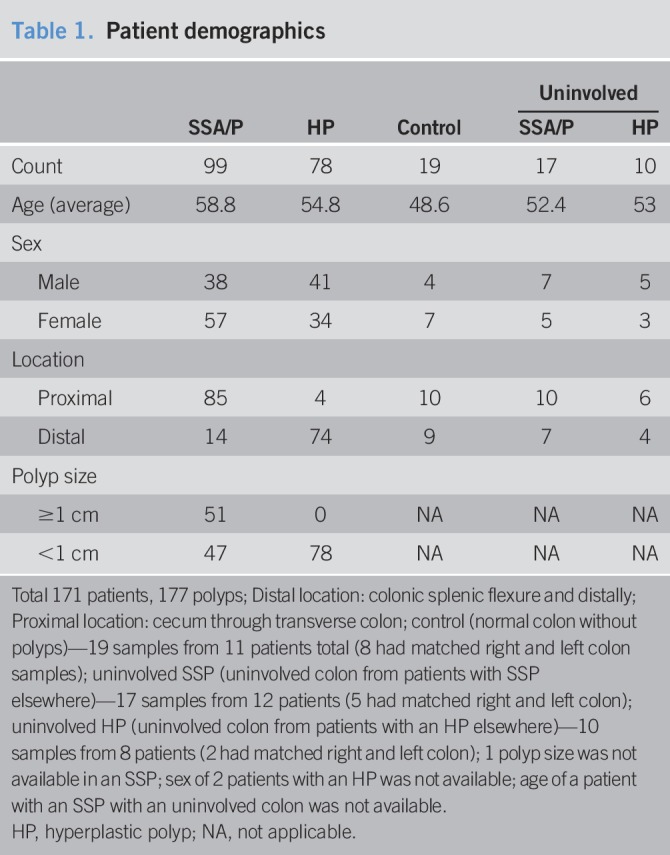
Patient demographics

### Differential RNA expression by polyp size and location

**Figure 2.**
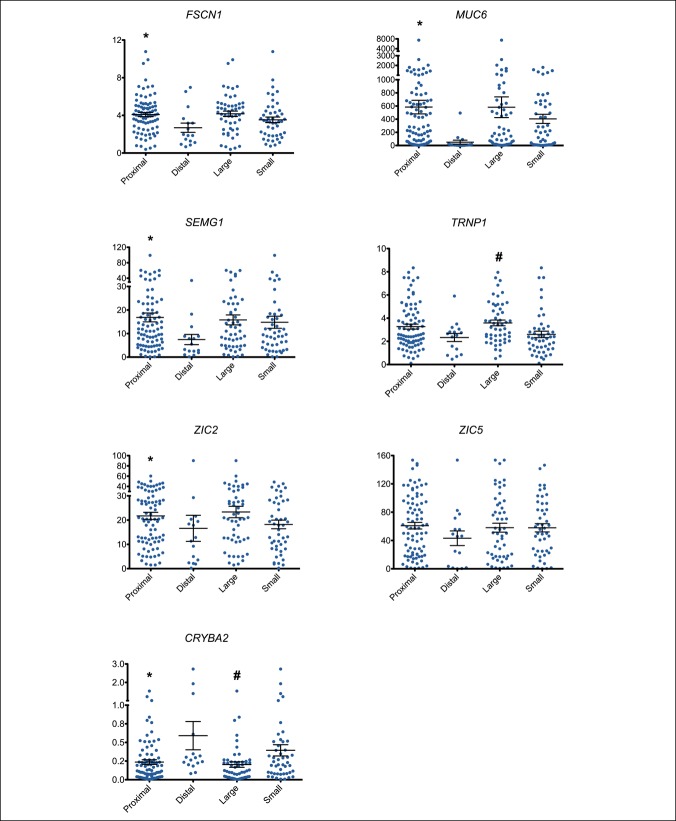
RNA expression of the 7-gene panel based on the size and location of SSA/Ps (n = 99), graphing each gene individually (*FSCN1*, *MUC6*, *SEMG1*, *TRNP1*, *ZIC2*, *ZIC5*, and *CRYBA2*). Y axis shows the fold change in expression relative to the mean of HP expression. X axis shows each of the 4 groups (proximal-proximal to splenic flexure; large >/ = 10 mm). Mean ± SE bars are presented for each of the 4 groups. Mann-Whitney *U*-test statistics in GraphPad Prism 5.0d version software were used to determine statistical significance between sample groups. The expression of *FSCN1*, *MUC6*, *SEMG1*, *ZIC2*, and *CRYBA2* was statistically significant (*P < 0.05) by location. The expression of *TRNP1* and *CRYBA2* was statistically significant (#*P* < 0.05) by size. HP, hyperplastic polyp.

Previous studies show that both polyp size and location may affect the level of gene expression (11,21). Therefore, we evaluated the effect of polyp size and location on the magnitude of gene expression change in each SSA/P (Figure 2). SSA/Ps from the right colon showed significantly higher expression of *FSCN1*, *MUC6*, *SEMG1*, and *ZIC2* compared with SSA/Ps from the left colon. SSA/Ps from the right colon also showed significantly lower expression of *CRYBA2*. However, in all cases except for MUC6, this change in expression was less than 2.5-fold. In addition, large SSA/Ps (≥1 cm) showed higher expression of *TRNP1* (1.4-fold) and lower expression of *CRYBA2* (2-fold) compared with small SSA/Ps. In short, the expression of 2 of 7 gene markers was not significantly influenced by colon location and expression of 5 of 7 was not influenced by polyp size.

### Three-point criteria for estimating sensitivity and specificity

Significant interobserver variability exists among pathologists diagnosing SSA/Ps and HPs. Because of this histological overlap and the influence of polyp size and location on gene expression, we reevaluated our serrated polyp samples using a 3-point criteria system as described in methods. Results were evaluated according to the area under the curve (ROC) using training and test set samples (Table 2). Table 2, (see Supplementary Digital Content 3, http://links.lww.com/CTG/A123), shows the 0–3 criteria designations for the 51 and 126 polyps in the training and test sets, respectively. The training set constituted polyps fulfilling either none or all of the 3 criteria (29 classical HPs, 22 classical SSA/Ps). Polyps fulfilling 1 and 2 of 3 criteria were part of the test set (1/3 criteria, n = 18; 2/3 criteria, n = 38). In addition, in the test set, 43 polyps met zero criteria (classical HPs) and 27 fulfilled all 3 criteria (classical SSA/Ps). The 7-gene panel showed 100% sensitivity and 97% specificity to identify serrated polyps that met all 3 criteria in the training set (Table 2). In the test set, 85.2% of serrated polyps that fulfilled all 3 criteria were positive for the gene signature (Table 2). For serrated polyps fulfilling 2 of 3 criteria, the gene signature showed 92.1% positivity, suggesting these polyps were predominantly SSA/P phenotype. Polyps fulfilling 1 of 3 had a lower positivity (55.6%), suggesting these polyps represented both SSA/P and HP phenotypes. Polyps that met 0 of 3 criteria were 11.6% positive, suggesting most of these had strong HP phenotypes. As more of the 3 criteria were met, the positivity of the gene panel increased, with little to no difference between polyps that fulfilled 2 or 3 of the 3 criteria.

**Table 2.**
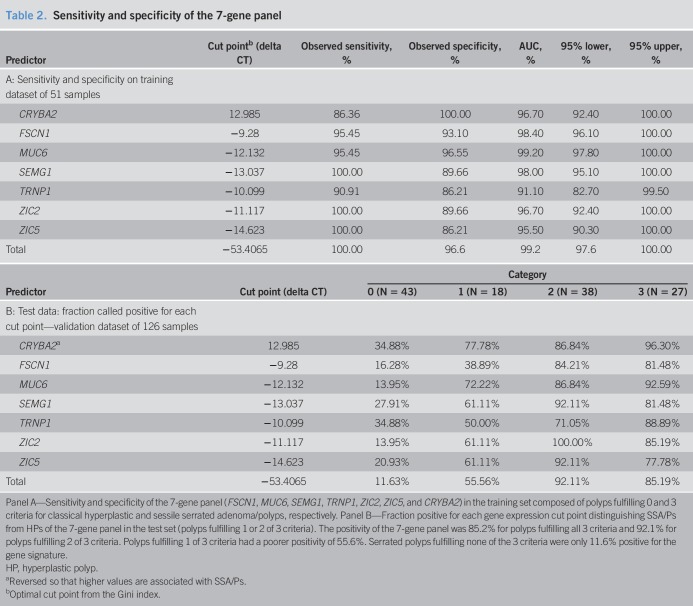
Sensitivity and specificity of the 7-gene panel

The sensitivity and specificity analysis on the test data is an unbiased estimate. The specificity on category 0 was 88.4%. The combined sensitivity on categories 2 and 3 was 89.2%. The gene panel was ambivalent on criteria 1 of 3 polyps. The approximate 10% difference between training and test data specificity is “overfitting.” Of the 3 criteria, polyp size contributed the least for the classification of SSA/P. Each polyp's initial histopathological diagnosis was compared with its RT-qPCR results (see Table 2, Supplementary Digital Content 2, http://links.lww.com/CTG/A122). Eighteen of 126 polyps (14%) showed discordant qPCR diagnoses compared with the initial histopathological diagnosis. Fold change values for the 7-gene panel for all serrated polyps using the 3 criteria system are shown in Figure 2, (see Supplementary Digital Content 4, http://links.lww.com/CTG/A119). *P* values for each group comparison are shown in Table 3, (see Supplementary Digital Content 5, http://links.lww.com/CTG/A124).

### Comparison of small, left-sided SSA/Ps and HPs

**Figure 3.**
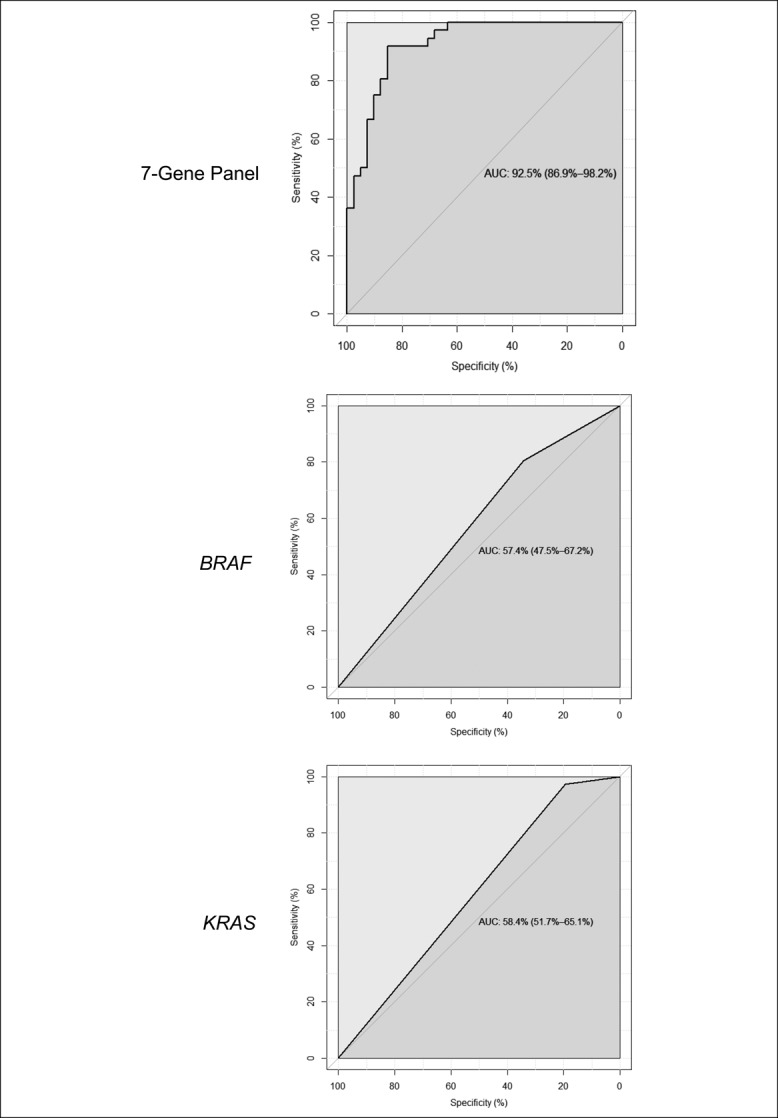
ROC curves of the 7-gene expression panel (*FSCN1*, *MUC6*, *SEMG1*, *TRNP1*, *ZIC2*, *ZIC5*, and *CRYBA2*) (**a**), and *BRAF* (**b**) and *KRAS* (**c**) mutation data, showing relative sensitivity and specificity in distinguishing SSA/Ps (n = 36) and HPs (n = 41). Serrated polyps fulfilling 2 or 3 of 3 criteria were considered SSA/Ps, and serrated polyps fulfilling 0 or 1 of 3 criteria were considered HPs. The 7-gene panel had high sensitivity and specificity (AUC 92.5%) in differentiating SSA/Ps and HPs, whereas *BRAF* and *KRAS* mutations did not (AUCs 57.4% and 58.4%, respectively. AUC, area under the curve; ROC, receiver operator characteristic; SSA/P, sessile serrated adenoma/polyp.

To determine whether polyp morphology alone was sufficient to identify differential expression of our 7-gene panel in the left colon, we compared small, left-sided SSA/Ps and HPs (see Figure 3, Supplementary Digital Content 6, http://links.lww.com/CTG/A120). All 7 genes showed statistically significant differential expression between criteria 1 of 3 SSA/Ps (n = 14) and criteria 0 of 3 HPs (n = 72). It should be noted that HPs showing the largest fold change (more SSA/P like) and SSA/Ps showing the smallest fold change (more HP like) exhibit those changes across all 7 genes.

### *BRAF* and *KRAS* mutation analysis

**Figure 4.**
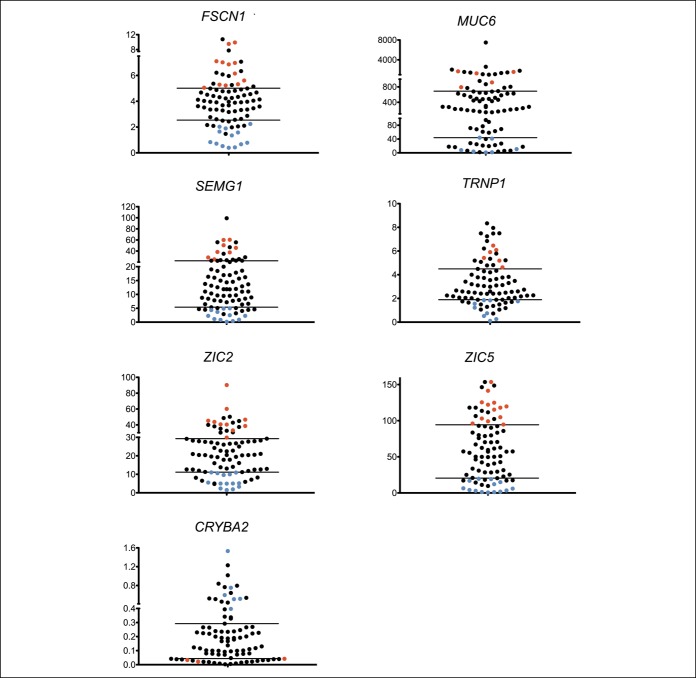
Range of fold change expression of the 7-gene panel (*FSCN1*, *MUC6*, *SEMG1*, *TRNP1*, *ZIC2*, *ZIC5*, and *CRYBA2*) in serrated polyps fulfilling 2 or 3 of the 3 criteria (n = 87). Red and blue dots show polyps with serrated sessile polyp-type expression of a minimum of 4 of 7 genes in the top or bottom 25% for all polyps, respectively. Lines separate the 25% highest and lowest-expressing polyp samples for each gene. Y axis depicts fold change from the mean of 0 of 3 criteria polyps (n = 72), and x axis shows individual SSA/P fold change values.

Because *BRAF* and *KRAS* mutations are common in serrated colon polyps, we determined the incidence of these mutations in a subset of 77 serrated polyps using the abovementioned 3-criteria system (Table 3). The incidence of *BRAF* mutation was high among all serrated polyp groups, with the highest incidence (76%–91%) occurring in serrated polyps fulfilling 2 or more of the 3 criteria (likely SSA/Ps). Serrated polyps fulfilling zero of 3 criteria (classical HPs) showed a 63% incidence of BRAF mutation. Similar to previous studies, *BRAF* mutation does not appear to be a reliable discriminator of SSA/Ps and HPs. By contrast, *KRAS* mutations were less frequent (range 0%–26%) in serrated polyps, with most mutations (88%) occurring in polyps fulfilling zero of the 3 criteria (classical HPs). *KRAS* mutations may have some value in identifying a subset of HPs although our sample size is too small to determine this. For comparison purposes, we evaluated the ability of our 7-gene panel to discriminate between the same 77 serrated polyps. Our gene panel demonstrated high sensitivity and specificity, whereas *BRAF* and *KRAS* mutations showed poor sensitivity and specificity, as shown in the ROC analysis (Figure 3). Finally, we determined whether the presence or absence of *BRAF* and *KRAS* mutations influenced the expression of our 7-gene panel. In most cases, *BRAF* mutation increased the level of fold change of each gene, especially in polyps meeting zero or one of the 3 criteria (likely HPs) (see Figure 4 and Table 4, Supplementary Digital Content 7, http://links.lww.com/CTG/A125).

**Table 3.**
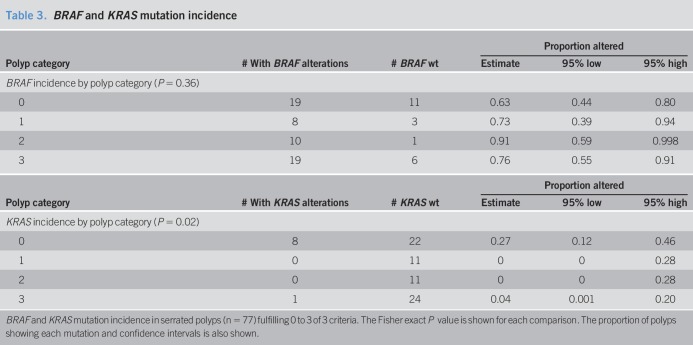
*BRAF* and *KRAS* mutation incidence

### Clinical parameters and gene expression

We observed a wide range in RNA expression among the 87 serrated polyp samples fulfilling 2 or 3 of the 3 criteria (likely SSA/Ps) for each of the 7 genes (Figure 4). For example, *FSCN1* fold expression changes ranged between <1 and >10-fold among the 87 serrated polyp samples. To identify clinical parameters that might help explain these differences in gene expression, we reviewed the clinical data for the 87 patients' serrated polyp samples with very high (top 25%) or very low (bottom 25%) fold changes for each gene (Figure 4, see Table 5, Supplementary Digital Content 8, http://links.lww.com/CTG/A126). Clinical data from 12 patients who showed the strongest fold changes of 4 or more of 7 genes in the panel and 15 patients with the weakest fold changes of 4 or more of the 7 genes were compared (Table 4). Although not statistically significant, patients with samples with the highest fold changes were more often older (mean age 63 vs 53 years) or were women (7 women vs 5 men). Interestingly, all (6 of 6) patients showing the highest differential expression, who underwent follow-up colonoscopy, had SSA/Ps or proximal HPs on their subsequent examination. By contrast, only 3 of 7 (43%) patients with the lowest differential expression, who underwent surveillance colonoscopy, had SSA/Ps. Comparing these 2 groups using the Fisher exact test results in a *P* value = 0.07. Time to follow-up colonoscopy ranged between 1 and 3 years.

**Table 4.**
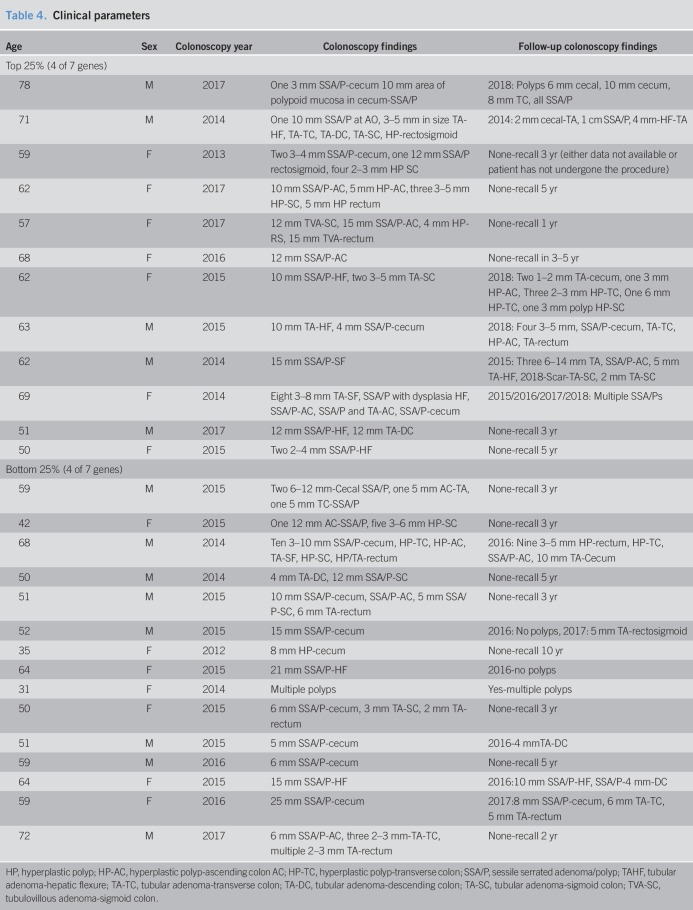
Clinical parameters

## DISCUSSION

Differentiating SSA/Ps from HPs can be challenging in clinical practice. Significant interobserver and intraobserver variability exists among pathologists (7–10). At present, no gold standard exists for molecular or histological diagnoses of SSA/Ps. There is a need for robust molecular markers that can accurately identify SSA/Ps and HPs. The RT-qPCR results presented here using FFPE tissue support our 7-gene panel as a reproducible molecular tool to distinguish SSA/Ps from HPs (11,22). Based on the RT-qPCR panel test results, approximately 1 in 7 HPs were reclassified as SSA/Ps and 1 in 7 SSA/Ps were reclassified as HPs when compared with the original histopathological diagnoses. This is significant because our cohort originates from a large tertiary care academic center with highly skilled and subspecialized GI pathologists.

In clinical practice, an average-risk patient with only HPs requires colonoscopy surveillance every 10 years, whereas those with SSA/Ps require follow-up colonoscopy every 3–5 years (6). Accurate detection of SSA/Ps is critical in routine clinical practice to avoid interval cancers resulting from inadequate follow-up surveillance colonoscopies. More accurate diagnoses will also better inform proper surveillance intervals in large cohort studies with long-term follow-up. On the other hand, misclassifying an HP as an SSA/P can lead to unnecessary, invasive, and expensive colonoscopy procedures. A gene expression analysis tool has limitations for routine use, including cost and false positives or false negatives; nonetheless, it may be a valuable adjunct to histological diagnoses in borderline or overlapping cases. Thus, a reproducible and accurate biomarker could be especially useful for diagnosing SSA/Ps with characteristics that overlap with HPs (1 of our 3 criteria). Our data suggest that some small left-sided SSA/Ps may be clinically significant and pose some risk for development into colon cancer, given their distinctive gene expression differences. Histopathological diagnosis could be guided by the magnitude of gene expression differences in criteria—1-of-3 serrated polyps. Long-term clinical follow-up data would be required to definitively answer this question. It is possible that different surveillance colonoscopy intervals could be recommended for patients with right- vs left-sided SSA/Ps. Such a tool could also assist serrated polyp research where accurate classification requires a gold standard. Our clinical data suggest that patients with high expression differences in our 7-gene panel are more likely to develop more SSA/Ps in the future. In short, our 7-gene panel cannot only differentiate SSA/Ps from HPs but may also be used as a predictive biomarker of SSA/P development. Larger multicenter studies are needed to determine the clinical impact of these biomarkers.

Our molecular data suggest that a polyp's fulfillment of 2 of 3 criteria is equally effective in classifying it as an SSA/P as fulfillment of 3 of 3 criteria. Previous studies have emphasized that large and right-sided serrated polyps are more likely to be SSA/Ps, and this is consistent with our gene panel results (6). Serrated colon cancers likely to develop from SSA/Ps are often right sided. Our gene panel results suggest that colon location may be a better predictor of SSA/Ps than polyp size measured during colonoscopy.

*BRAF* and *KRAS* mutations are common in serrated polyps (17,19,20,23). Multiple studies have shown a high level of *BRAF* mutation in both SSA/Ps and HPs. Some of these studies indicated higher *BRAF* mutation in a histologic subset of HPs called microvesicular HPs (17,20,23). Microvesicular HP usage in clinical practice remains uncertain and is not used to describe HPs in routine pathology practice (6). The HP cohort in our study largely comprised small polyps that were mostly from the left colon, and a considerable number were positive for *BRAF* mutation. We know from the natural history of polyps that most of the small, rectosigmoid HPs are benign and may not progress into cancer (24). The American Society of Gastrointestinal Endoscopy (ASGE) PIVI management guide states that small, left-sided HPs may be left *in situ* if they are considered nonadenomatous with a negative predictive value of >90% (25). In short, evidence suggests that *BRAF* mutation is not a reliable marker for differentiation of SSA/Ps from HPs and is unable to explain the biology of an SSA/P and its progression to neoplasia in its entirety.

Serrated pathway cancers have been shown to carry higher disease-specific mortality and are considered to be aggressive (26,27). CIMP-high tumors may respond poorly to the standard 5-fluorouracil–based adjuvant therapy for colorectal cancer (28). Serrated pathway cancers have also been indicated to carry a poor prognosis (29–31). Recent studies have shown an increased risk of interval or missed colon cancer (diagnosed within 6 months to 5 years after colonoscopy) in patients with SSA/Ps. These cancers tend to be in the proximal colon where SSA/Ps are usually found. Previous studies have suggested the roles of microsatellite instability and CpG island methylation in interval colon cancers, and both phenomena have been indicated in cancers arising from the serrated pathway (32,33). Limited data exist about the underlying genes playing a role in the serrated pathway, and no known germline mutations have been identified in patients with serrated polyposis syndrome. Hence, understanding the association of novel gene signatures of SSA/P and the progression from SSA/P to colorectal cancer remains critical. Such understanding will be crucial in designing management approaches and therapies for serrated pathway cancers.

In conclusion, we present new diagnostic data that confirm a recently described 7-gene panel that can be applied to FFPE samples using quantitative RT-PCR to differentiate SSA/Ps from HPs with high sensitivity and specificity. Because RT-qPCR was performed on easily accessible, archived, FFPE samples, this methodology has potential within clinical practice and to standardize research into the serrated pathway of colon cancer. This study provides further evidence of gene expression differences between the 2 major serrated colon polyp subtypes. These genes may have functional roles in the serrated pathway, and mechanistic studies are needed to understand their importance in serrated neoplastic progression.

## CONFLICTS OF INTEREST

**Guarantor of the article:** Priyanka Kanth, MD, MSCI.

**Specific author contributions:** P.K., M.P.B., M.W.H., and D.A.D.: conceived and designed the experiments. M.W.H., R.Y., S.P., and D.A.D.: performed the experiments. P.K., M.P.B., K.M.B., M.W.H., R.Y., and D.A.D.: analyzed the data. P.K., K.E.B., M.P.B., and D.A.D.: contributed samples/reagents/materials/tools. P.K., K.E.B., M.P.B., K.M.B., M.W.H., P.S.B., and D.A.D.: manuscript preparation.

**Financial support:** This study was supported by NIH NCI 1R21CA191507 (D.A.D. and P.K.).

**Potential competing interests:** Patent application submitted (U.S. Patent Application No.: 62/303,133) on 7-gene panel (D.A.D. and P.K.). We declare no competing interests, financial or otherwise, associated with this work for other coauthors.

**Study Highlights**

**WHAT IS KNOWN**

✓ Sessile serrated adenoma/polyps (SSA/Ps) are precursor lesions accounting for up to 30% of colon cancers.
✓ Significant interobserver variability exists among pathologists diagnosing SSA/Ps and HPs because of overlapping histological features between these polyp subtypes.
✓ Better diagnostic markers are needed to accurately differentiate SSA/Ps and HPs to improve clinical care and research in the serrated pathway of colon cancer.
✓ *BRAF* mutation plays a role in the serrated pathway to cancer but has limited utility in accurate identification of SSA/Ps.

**WHAT IS NEW HERE**

✓ We report a novel gene expression panel that can be used as a companion diagnostic tool with histology to improve the distinction of serrated polyps.
✓ The magnitude of RNA expression for most panel genes was not significantly influenced by polyp size. Based on the gene panel expression, small SSA/Ps (<10 mm) may pose a risk similar to that of large SSA/Ps.
✓ This gene panel performs better than *BRAF* mutation in identification of SSA/Ps.

**TRANSLATIONAL IMPACT**

✓ This study provides clinical validation of previously published RNA markers of SSA/Ps and supports the importance of these genes in colon cancer development from SSA/Ps.
✓ The magnitude of gene expression within an SSA/P may have predictive value to indicate a patient's risk for developing future SSA/Ps.

## Supplementary Material

SUPPLEMENTARY MATERIAL
