# Supplementary material for: Molecular Biomarkers of Sessile Serrated Adenoma/Polyps
Source: Clin Transl Gastroenterol. 2019 Nov 26;10(12):e00104. doi: 10.14309/ctg.0000000000000104 (PMC6970553; doi:10.14309/ctg.0000000000000104)
Supplement: SUPPLEMENTARY MATERIAL [file ct9-10-e00104-s001.pdf]

Supplemental Table 1

	Fold Change		
Gene	vs HPs	vs Uninvolved	vs Control
CRYBA2	-4.85	-3.73	-4.33
FSCN1	3.16	3.73	3.25
MUC6	17.28	240.78	3485.29
SEMG1	4.93	29.89	22.99
TRNP1	2.27	11.32	12.39
ZIC2	6.72	42.97	44.76
ZIC5	8.3	32.44	91.3

All 21 comparisons were statistically significant by Mann Whitney U-Test ($p < 0.0001$)

**Gene**	**Description**	**Function/Process**
CRYBA2	crystallin beta A2	protein binding, lens development
FSCN1	fascin actin-binding protein 1	actin filament binding, cell migration
MUC6	mucin 6	extracellular matrix, maintenance of epithelium
SEMG1	semenogelin 1	protein binding, antibacterial humoral response
TRNP1	TMF1 regulated nuclear protein 1	DNA binding, cell cycle
ZIC2	Zic family member 2	DNA binding, cell differentiation
ZIC5	Zic family member 5	DNA binding, cell differentiation

**Gene**	**Relevance to colon polyps / cancer**
CRYBA2	unknown
FSCN1	tumor invasion
MUC6	epithelium integrity / organogenesis
SEMG1	unknown
TRNP1	highly expressed in right-sided colon cancers
ZIC2	regulates WNT signaling
ZIC5	regulates WNT signaling
