# Supplementary figures and images for: Molecular Biomarkers of Sessile Serrated Adenoma/Polyps
Source: Clin Transl Gastroenterol. 2019 Nov 26;10(12):e00104. doi: 10.14309/ctg.0000000000000104 (PMC6970553; doi:10.14309/ctg.0000000000000104)

## Supplemental Figure 1

### FFPE Sample Flow Diagram

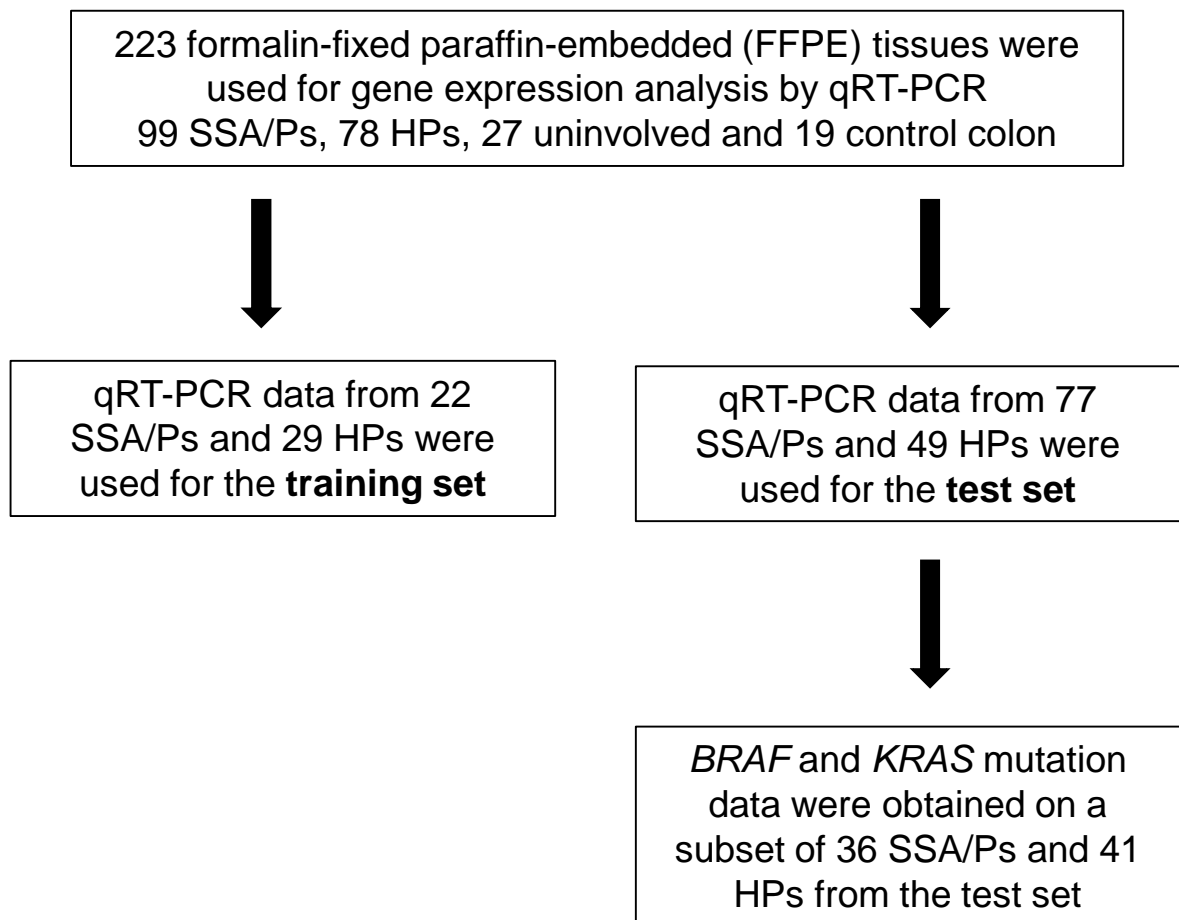

Supplement: SUPPLEMENTARY MATERIAL [file ct9-10-e00104-s002.pdf]

Supplemental Figure 2

FSCN1

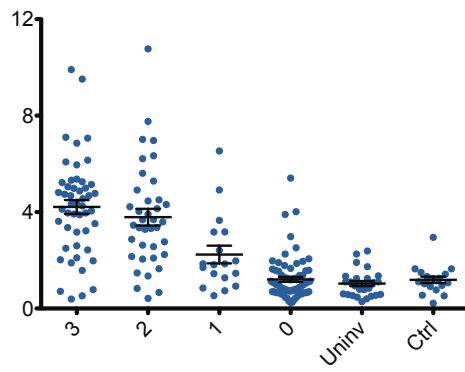

MUC6

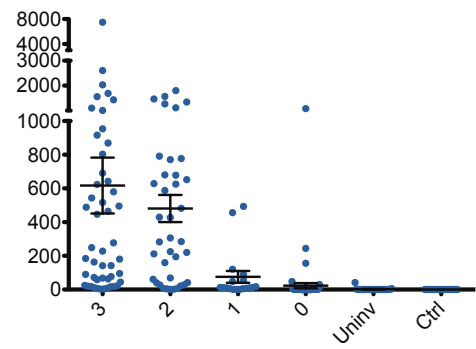

SEMG1

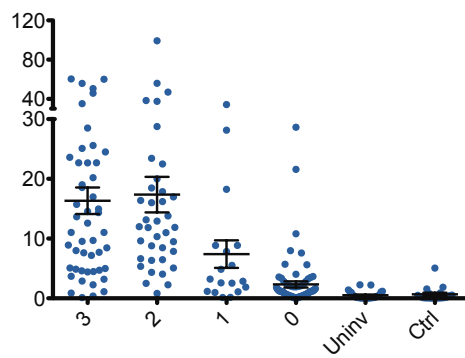

TRNP1

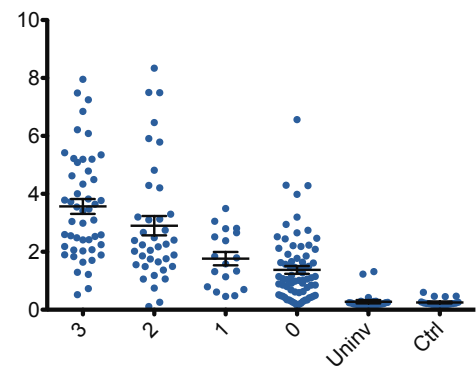

ZIC2

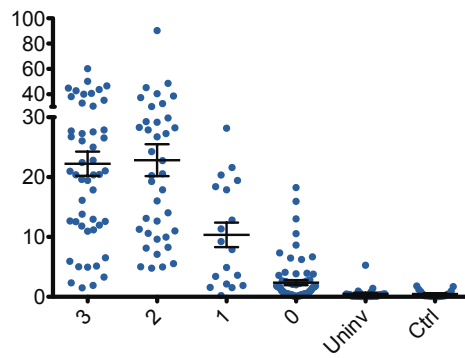

ZIC5

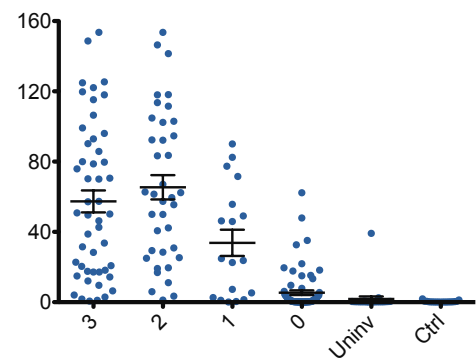

CRYBA2

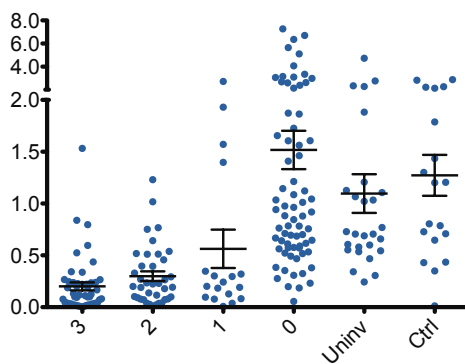

Supplement: SUPPLEMENTARY MATERIAL [file ct9-10-e00104-s003.pdf]

# Supplemental Figure 3

CRYBA2

FC=0.465  
p=.0029

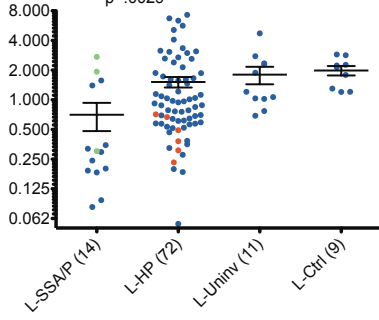

FSCN1

FC=2.115  
p=.0001

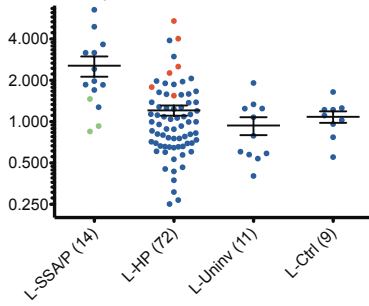

MUC6

FC=3.968  
p<.0001

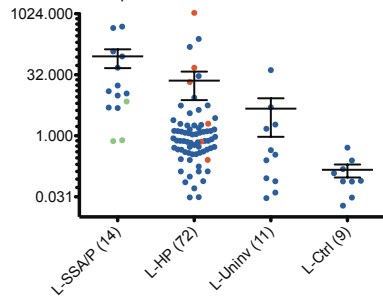

SEMG1

FC=3.796  
p=.0013

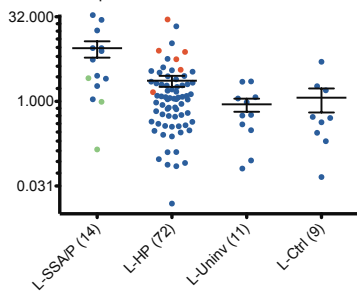

TRNP1

FC=1.466  
p=.0129

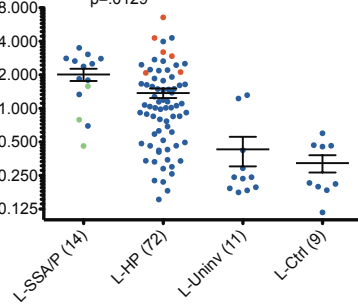

ZIC2

FC=5.163  
p<.0001

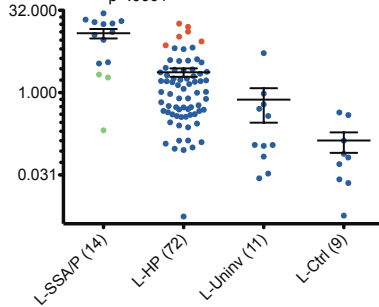

ZIC5

FC=7.618  
p=.0001

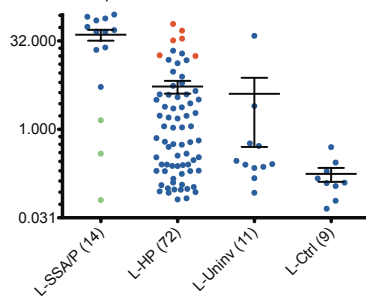

Supplement: SUPPLEMENTARY MATERIAL [file ct9-10-e00104-s005.pdf]

# Supplemental Figure 4

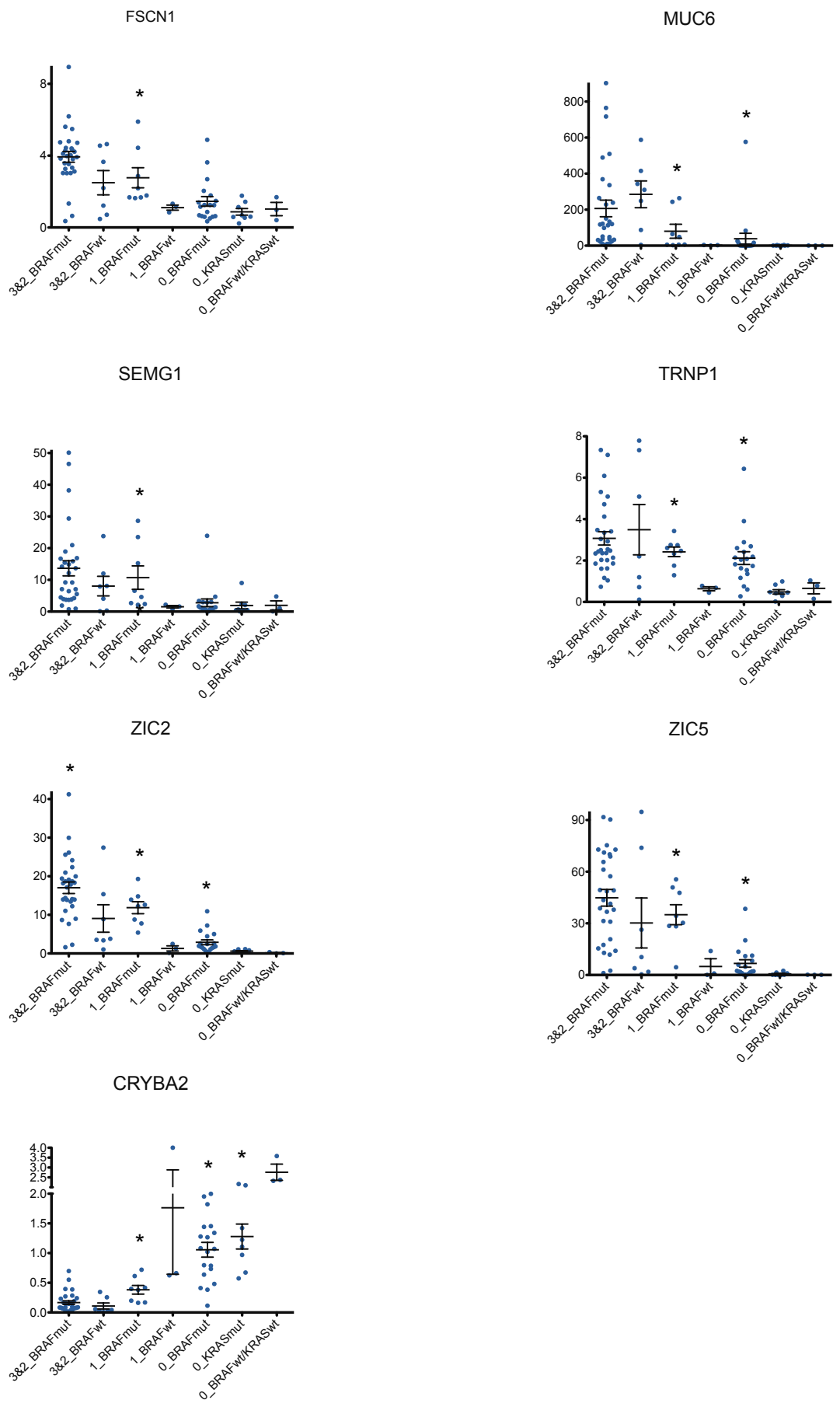

Supplement: SUPPLEMENTARY MATERIAL [file ct9-10-e00104-s007.pdf]
