# Supplementary material for: Molecular Biomarkers of Sessile Serrated Adenoma/Polyps
Source: Clin Transl Gastroenterol. 2019 Nov 26;10(12):e00104. doi: 10.14309/ctg.0000000000000104 (PMC6970553; doi:10.14309/ctg.0000000000000104)
Supplement: SUPPLEMENTARY MATERIAL [file ct9-10-e00104-s004.pdf]

Supplemental Table 3

Mann-Whitney p values for 4 comparisons to 'polyp 0' category -across all 223 samples

	0 vs 3	0 vs 2	0 vs 1	0 vs Ctrl
**FSCN1**	<0.0001	<0.0001	0.0009	0.4038
**MUC6**	<0.0001	<0.0001	<0.0001	<0.0001
**SEMG1**	<0.0001	<0.0001	0.0026	0.0003
**TRNP1**	<0.0001	<0.0001	0.0586	<0.0001
**ZIC2**	<0.0001	<0.0001	<0.0001	0.0002
**ZIC5**	<0.0001	<0.0001	<0.0001	0.0003
**CRYBA2**	<0.0001	<0.0001	<0.0001	0.8261

n for each of these tissue categories:

3	49
2	38
1	18
0	72
Ctrl	19
