# Supplementary material for: Molecular Biomarkers of Sessile Serrated Adenoma/Polyps
Source: Clin Transl Gastroenterol. 2019 Nov 26;10(12):e00104. doi: 10.14309/ctg.0000000000000104 (PMC6970553; doi:10.14309/ctg.0000000000000104)
Supplement: SUPPLEMENTARY MATERIAL [file ct9-10-e00104-s006.pdf]

FSCN1		p value
3&2_BRAFmut(n=29):	3.933	0.1288
3&2_BRAFwt(n=7):	2.492	
1_BRAFmut(n=8):	2.768	0.0121
1_BRAFwt(n=3):	1.103	
0_BRAFmut(n=19):	1.453	0.6323
0_KRASmut(n=8):	0.869	0.9212
0_BRAFwt(n=3):	1.024	

MUC6		p value
3&2_BRAFmut(n=29):	206.479	0.2805
3&2_BRAFwt(n=7):	285.015	
1_BRAFmut(n=8):	79.873	0.0485
1_BRAFwt(n=3):	2.272	
0_BRAFmut(n=19):	38.339	0.0445
0_KRASmut(n=8):	1.140	0.1939
0_BRAFwt(n=3):	0.231	

SEMG1		p value
3&2_BRAFmut(n=29):	13.646	0.2631
3&2_BRAFwt(n=7):	8.036	
1_BRAFmut(n=8):	10.707	0.0242
1_BRAFwt(n=3):	1.513	
0_BRAFmut(n=19):	2.770	0.9323
0_KRASmut(n=8):	1.912	1.000
0_BRAFwt(n=3):	1.951	

TRNP1		p value
3&2_BRAFmut(n=29):	3.069	0.7796
3&2_BRAFwt(n=7):	3.489	
1_BRAFmut(n=8):	2.421	0.0121
1_BRAFwt(n=3):	0.636	
0_BRAFmut(n=19):	2.117	0.0353
0_KRASmut(n=8):	0.485	0.6303
0_BRAFwt(n=3):	0.655	

ZIC2		p value
3&2_BRAFmut(n=29):	17.023	0.0415
3&2_BRAFwt(n=7):	9.052	
1_BRAFmut(n=8):	11.854	0.0121
1_BRAFwt(n=3):	1.298	
0_BRAFmut(n=19):	2.891	0.0098
0_KRASmut(n=8):	0.596	0.0848
0_BRAFwt(n=3):	0.140	

ZIC5		p value
3&2_BRAFmut(n=29):	44.847	0.1872
3&2_BRAFwt(n=7):	30.202	
1_BRAFmut(n=8):	35.014	0.0242
1_BRAFwt(n=3):	4.941	
0_BRAFmut(n=19):	6.696	0.0074
0_KRASmut(n=8):	0.650	0.6303
0_BRAFwt(n=3):	0.080	

CRYBA2		p value
3&2_BRAFmut(n=29):	0.166	0.1391
3&2_BRAFwt(n=7):	0.109	
1_BRAFmut(n=8):	0.382	0.0485
1_BRAFwt(n=3):	1.763	
0_BRAFmut(n=19):	1.056	0.0074
0_KRASmut(n=8):	1.278	0.0121
0_BRAFwt(n=3):	2.759	
