# Supplementary material for: Molecular Biomarkers of Sessile Serrated Adenoma/Polyps
Source: Clin Transl Gastroenterol. 2019 Nov 26;10(12):e00104. doi: 10.14309/ctg.0000000000000104 (PMC6970553; doi:10.14309/ctg.0000000000000104)
Supplement: SUPPLEMENTARY MATERIAL [file ct9-10-e00104-s008.docx]

**Supplemental Table 2. Training and Test Set Samples**

| **51-polyp training set** | |  |  |  |  |  |
| --- | --- | --- | --- | --- | --- | --- |
| Original  Histopathology Diagnosis | Number Samples | Criteria | | | Score | RT-qPCR Diagnosis |
|  |  | Polyp Size | Location | Morphology |  |  |
| SSA/P | 22 | Large | Right | SSA/P-like | 3 | SSA/P |
| HP | 29 | Small | Left | HP | 0 | HP |
|  |  |  |  |  |  |  |
| **126-polyp testing set** | |  |  |  |  |  |
| Sample Name | Original  Histopathology Diagnosis | Criteria | | | Score | RT-qPCR Diagnosis |
|  |  | Polyp Size | Location | Morphology |  |  |
| SSA/P-1 | SSA/P | large | right | SSA/P-like | 3 | SSA/P |
| SSA/P-2 | SSA/P | large | right | SSA/P-like | 3 | SSA/P |
| SSA/P-3 | SSA/P | large | right | SSA/P-like | 3 | SSA/P |
| SSA/P-4 | SSA/P | large | right | SSA/P-like | 3 | SSA/P |
| SSA/P-5 | SSA/P | large | right | SSA/P-like | 3 | HP |
| SSA/P-6 | SSA/P | large | right | SSA/P-like | 3 | SSA/P |
| SSA/P-7 | SSA/P | large | right | SSA/P-like | 3 | SSA/P |
| SSA/P-8 | SSA/P | large | right | SSA/P-like | 3 | SSA/P |
| SSA/P-9 | SSA/P | large | right | SSA/P-like | 3 | HP |
| SSA/P-10 | SSA/P | large | right | SSA/P-like | 3 | SSA/P |
| SSA/P-11 | SSA/P | large | right | SSA/P-like | 3 | SSA/P |
| SSA/P-12 | SSA/P | large | right | SSA/P-like | 3 | SSA/P |
| SSA/P-13 | SSA/P | large | right | SSA/P-like | 3 | SSA/P |
| SSA/P-14 | SSA/P | large | right | SSA/P-like | 3 | SSA/P |
| SSA/P-15 | SSA/P | large | right | SSA/P-like | 3 | SSA/P |
| SSA/P-16 | SSA/P | large | right | SSA/P-like | 3 | HP |
| SSA/P-17 | SSA/P | large | right | SSA/P-like | 3 | SSA/P |
| SSA/P-18 | SSA/P | large | right | SSA/P-like | 3 | SSA/P |
| SSA/P-19 | SSA/P | large | right | SSA/P-like | 3 | SSA/P |
| SSA/P-20 | SSA/P | large | right | SSA/P-like | 3 | SSA/P |
| SSA/P-21 | SSA/P | large | right | SSA/P-like | 3 | SSA/P |
| SSA/P-22 | SSA/P | large | right | SSA/P-like | 3 | SSA/P |
| SSA/P-23 | SSA/P | large | right | SSA/P-like | 3 | SSA/P |
| SSA/P-24 | SSA/P | large | right | SSA/P-like | 3 | SSA/P |
| SSA/P-25 | SSA/P | large | right | SSA/P-like | 3 | SSA/P |
| SSA/P-26 | SSA/P | large | right | SSA/P-like | 3 | SSA/P |
| SSA/P-27 | SSA/P | large | right | SSA/P-like | 3 | HP |
| SSA/P-28 | SSA/P | small | right | SSA/P-like | 2 | SSA/P |
| SSA/P-29 | SSA/P | small | right | SSA/P-like | 2 | SSA/P |
| SSA/P-30 | SSA/P | small | right | SSA/P-like | 2 | SSA/P |
| SSA/P-31 | SSA/P | small | right | SSA/P-like | 2 | SSA/P |
| SSA/P-32 | SSA/P | small | right | SSA/P-like | 2 | SSA/P |
| SSA/P-33 | SSA/P | small | right | SSA/P-like | 2 | SSA/P |
| SSA/P-34 | SSA/P | small | right | SSA/P-like | 2 | SSA/P |
| SSA/P-35 | SSA/P | small | right | SSA/P-like | 2 | SSA/P |
| SSA/P-36 | SSA/P | small | right | SSA/P-like | 2 | SSA/P |
| SSA/P-37 | SSA/P | small | right | SSA/P-like | 2 | SSA/P |
| SSA/P-38 | SSA/P | small | right | SSA/P-like | 2 | SSA/P |
| SSA/P-39 | SSA/P | small | right | SSA/P-like | 2 | SSA/P |
| SSA/P-40 | SSA/P | small | right | SSA/P-like | 2 | SSA/P |
| SSA/P-41 | SSA/P | small | right | SSA/P-like | 2 | SSA/P |
| SSA/P-42 | SSA/P | small | right | SSA/P-like | 2 | SSA/P |
| SSA/P-43 | SSA/P | small | right | SSA/P-like | 2 | SSA/P |
| SSA/P-44 | SSA/P | small | right | SSA/P-like | 2 | SSA/P |
| SSA/P-45 | SSA/P | small | right | SSA/P-like | 2 | SSA/P |
| SSA/P-46 | SSA/P | small | right | SSA/P-like | 2 | SSA/P |
| SSA/P-47 | SSA/P | small | right | SSA/P-like | 2 | SSA/P |
| SSA/P-48 | SSA/P | small | right | SSA/P-like | 2 | SSA/P |
| SSA/P-49 | SSA/P | small | right | SSA/P-like | 2 | SSA/P |
| SSA/P-50 | SSA/P | small | right | SSA/P-like | 2 | SSA/P |
| SSA/P-51 | SSA/P | small | right | SSA/P-like | 2 | SSA/P |
| SSA/P-52 | SSA/P | small | right | SSA/P-like | 2 | SSA/P |
| SSA/P-53 | SSA/P | small | right | SSA/P-like | 2 | SSA/P |
| SSA/P-54 | SSA/P | small | right | SSA/P-like | 2 | SSA/P |
| SSA/P-55 | SSA/P | small | right | SSA/P-like | 2 | SSA/P |
| SSA/P-56 | SSA/P | small | right | SSA/P-like | 2 | SSA/P |
| SSA/P-57 | SSA/P | small | right | SSA/P-like | 2 | SSA/P |
| SSA/P-58 | SSA/P | small | right | SSA/P-like | 2 | SSA/P |
| SSA/P-59 | SSA/P | small | right | SSA/P-like | 2 | SSA/P |
| SSA/P-60 | SSA/P | large | left | SSA/P-like | 2 | SSA/P |
| SSA/P-61 | SSA/P | large | left | SSA/P-like | 2 | HP |
| SSA/P-62 | SSA/P | large | left | SSA/P-like | 2 | SSA/P |
| SSA/P-63 | SSA/P | small | left | SSA/P-like | 1 | HP |
| SSA/P-64 | SSA/P | small | left | SSA/P-like | 1 | SSA/P |
| SSA/P-65 | SSA/P | small | left | SSA/P-like | 1 | SSA/P |
| SSA/P-66 | SSA/P | small | left | SSA/P-like | 1 | HP |
| SSA/P-67 | SSA/P | small | left | SSA/P-like | 1 | SSA/P |
| SSA/P-68 | SSA/P | small | left | SSA/P-like | 1 | SSA/P |
| SSA/P-69 | SSA/P | small | left | SSA/P-like | 1 | SSA/P |
| SSA/P-70 | SSA/P | small | left | SSA/P-like | 1 | HP |
| SSA/P-71 | SSA/P | small | left | SSA/P-like | 1 | SSA/P |
| SSA/P-72 | SSA/P | small | left | SSA/P-like | 1 | HP |
| SSA/P-73 | SSA/P | small | left | SSA/P-like | 1 | SSA/P |
| SSA/P-74 | SSA/P | small | left | SSA/P-like | 1 | SSA/P |
| SSA/P-75 | SSA/P | small | right | HP | 1 | SSA/P |
| SSA/P-76 | SSA/P | small | right | HP | 1 | HP |
| SSA/P-77 | SSA/P | small | left | HP | 0 | HP |
| HP-1 | HP | small | right | SSA/P-like | 2 | HP |
| HP-2 | HP | small | right | SSA/P-like | 2 | SSA/P |
| HP-3 | HP | large | left | SSA/P-like | 2 | HP |
| HP-4 | HP | small | right | HP | 1 | HP |
| HP-5 | HP | small | right | HP | 1 | HP |
| HP-6 | HP | small | left | SSA/P-like | 1 | HP |
| HP-7 | HP | small | left | SSA/P-like | 1 | SSA/P |
| HP-8 | HP | small | left | HP | 0 | HP |
| HP-9 | HP | small | left | HP | 0 | HP |
| HP-10 | HP | small | left | HP | 0 | HP |
| HP-11 | HP | small | left | HP | 0 | HP |
| HP-12 | HP | small | left | HP | 0 | HP |
| HP-13 | HP | small | left | HP | 0 | SSA/P |
| HP-14 | HP | small | left | HP | 0 | HP |
| HP-15 | HP | small | left | HP | 0 | HP |
| HP-16 | HP | small | left | HP | 0 | HP |
| HP-17 | HP | small | left | HP | 0 | HP |
| HP-18 | HP | small | left | HP | 0 | HP |
| HP-19 | HP | small | left | HP | 0 | HP |
| HP-20 | HP | small | left | HP | 0 | HP |
| HP-21 | HP | small | left | HP | 0 | HP |
| HP-22 | HP | small | left | HP | 0 | HP |
| HP-23 | HP | small | left | HP | 0 | HP |
| HP-24 | HP | small | left | HP | 0 | SSA/P |
| HP-25 | HP | small | left | HP | 0 | SSA/P |
| HP-26 | HP | small | left | HP | 0 | HP |
| HP-27 | HP | small | left | HP | 0 | HP |
| HP-28 | HP | small | left | HP | 0 | HP |
| HP-29 | HP | small | left | HP | 0 | HP |
| HP-30 | HP | small | left | HP | 0 | HP |
| HP-31 | HP | small | left | HP | 0 | HP |
| HP-32 | HP | small | left | HP | 0 | HP |
| HP-33 | HP | small | left | HP | 0 | HP |
| HP-34 | HP | small | left | HP | 0 | HP |
| HP-35 | HP | small | left | HP | 0 | HP |
| HP-36 | HP | small | left | HP | 0 | HP |
| HP-37 | HP | small | left | HP | 0 | HP |
| HP-38 | HP | small | left | HP | 0 | HP |
| HP-39 | HP | small | left | HP | 0 | SSA/P |
| HP-40 | HP | small | left | HP | 0 | HP |
| HP-41 | HP | small | left | HP | 0 | HP |
| HP-42 | HP | small | left | HP | 0 | HP |
| HP-43 | HP | small | left | HP | 0 | HP |
| HP-44 | HP | small | left | HP | 0 | HP |
| HP-45 | HP | small | left | HP | 0 | HP |
| HP-46 | HP | small | left | HP | 0 | HP |
| HP-47 | HP | small | left | HP | 0 | SSA/P |
| HP-48 | HP | small | left | HP | 0 | HP |
| HP-49 | HP | small | left | HP | 0 | HP |
