# Supplementary material for: Molecular Biomarkers of Sessile Serrated Adenoma/Polyps
Source: Clin Transl Gastroenterol. 2019 Nov 26;10(12):e00104. doi: 10.14309/ctg.0000000000000104 (PMC6970553; doi:10.14309/ctg.0000000000000104)
Supplement: SUPPLEMENTARY MATERIAL [file ct9-10-e00104-s009.docx]

| **Supplemental Table 5** | | | | | | | | | | | | | | |
| --- | --- | --- | --- | --- | --- | --- | --- | --- | --- | --- | --- | --- | --- | --- |
| **Top 25% (4-of-7 gene)** | | | | | | | | | | | | | | |
| **Age** | **Sex** | **Ethnicity** | **Colonoscopy Indication** | **Colonoscopy year** | **Colonoscopy findings** | **Prior colonosocpy findings** | **Follow-up colonosocpy findings** | **Aspirin** | **DM** | **BMI** | **Vitamin D** | **Any cancer** | **Smoker** | **SPS** |
| 78 | M | Caucasian | Surveillance | 2017 | One 3 mm SSA/P- cecum 10mm area of polypoid mucosa in cecum- SSA/P | 2012: No polyps | 2018:,Polyps 6mm cecal, 10mm cecum, 8mm TC, All SSA/P | No | No | 19 | No | No | No | No |
| 71 | M | Caucasian | Screening | 2014 | One 10mm SSA/P at AO, 3-5mm in size TA-HF,TA-TC,TA-DC,TA-SC,HP-  Rectosigmoid | None | 2014: 2mm Cecal-TA, 1cm SSA/P, 4mm-HF-TA | No | No | 25 | No | No | Former | No |
| 59 | F | Caucasian | Screening | 2013 | Two 3 to 4 mm SSA/P-cecum, One 12mm SSA/P Rectosigmoid, Four 2- 3mm HP SC | None | None-Recall 3 years (either data not available or patient has not undergone procedure) | No | Yes | 45 | Yes(mvt) | Breast, Pituitary Adenoma | Former | No |
| 62 | F | Caucasian | Screening | 2017 | 10mm SSA/P -AC, 5mm HP- AC,  Three 3-5mm HP-SC, 5mm HP Rectum | 2005: Report not avail | None-Recall 5 years | No | No | 35 | Yes | No | No | No |
| 57 | F | Caucasian | Screening | 2017 | 12 mm TVA-SC, 15mm SSA/P-AC,  4mm HP-RS, 15mm TVA-Rectum | None | None-Recall 1 year | No | No | 29 | Yes | No | Yes | No |
| 68 | F | Caucasian | Surveillance | 2016 | 12mm SSA/P-AC | SSA/P-AC, SSA/P-  SF,HP-Rectum | None-Recall in 3-5 years | No | Prediabetes | 24 | Yes | No | No | No |
| 62 | F | Caucasian | Screening | 2015 | 10mmSSA/P-HF, Two 3-5mm TA-SC | None | 2018:Two 1 - 2 mm TA-  cecum, One 3 mm HP- AC,Three 2 to 3 mm HP-  TC,One 6 mm HP-TC, One 3 | No | No | 19 | Yes | No | No | No |
| 63 | M | Caucasian | Surveillance | 2015 | 10mm TA-HF, 4mm SSA/P-Cecum | 2002: One 5mm TA-  TC, 2009:Three small TA-TC, 2012-Four 2- | 2018:Four 3-5mm,SSA/P- Cecum, TA-TC,HP-AC,TA-  Rectum | Yes | No | 23 | Yes(mvt) | No | Former | No |
| 62 | M | Caucasian | Surveillance | 2014 | 15mm SSA/P-SF | Yes-Data not available | 2015:Three 6-14mm TA,SSA/P AC, 5mm TA-HF, 2018-Scar-TA- SC, 2mm TA-SC | Yes | No | 25 | Yes(mvt) | No | No | No |
| 69 | F | Caucasian | Surveillance | 2014 | Eight 3 to 8mm TA-SF, SSA/P with dysplasia HF,SSA/P- AC,SSA/P & TA- AC,SSA/P Cecum | 2010: 30mm TVA  with HGD, Cancer SC, 5mm TA-DC, Three 2- 3mm TA- SF,TA0TC,TA,AC,  Folds in HP-AC and HP-Cecum. 2010: | 2015/2016/2017/2018:  Multiple SSA/Ps, | Yes | No | 27 | Yes(mvt) | Sigmoid cancer (in polyp) | Former | Yes |
| 51 | M | Caucasian | Screening | 2017 | 12mm SSA/P-HF, 12mm TA-DC | None | None-Recall 3 years | No | No | 30 | No | No | Yes | No |
| 50 | F | Caucasian | Surveillance | 2015 | Two 2-4mm SSA/P HF | 2012: 9mm TA-  Rectum | None-Recall 5 years | No | No | 21 | No | No | No | No |
| **Bottom 25% (4-of-7 gene)** | | | | | | | | | | | | | | |
| 59 | M | Caucasian | Surveillance | 2015 | Two 6-12mm -Cecal SSA/P, One 5mm AC-TA, One 5mm TC-SSA/P | h/o polyps in prior scopes-data not available | None-Recall 3 years | No | Prediabetes | 26 | Yes | Basal cell cancer | No | No |
| 42 | F | Caucasian | Anemia | 2015 | One 12mm AC-SSA/P, Five 3-6mm HP-SC | None | None-Recall 3 years | None | No | 26 | No | No | Yes | No |
| 68 | M | Caucasian | Screening | 2014 | Ten 3 to 10 mm SSA/P-cecum,HP- TC, HP-AC,TA-SF,HP-SC,HP/TA-  Rectum. | None | 2016:Nine 3 to 5 mm HP- Rectum,HP-TC,SSA/P-AC,  10mm TA-Cecum. | Yes | No | 40 | No | Bladder | Former | No |
| 50 | M | Caucasian | Screening | 2014 | 4mm TA-DC, 12mm SSA/P-SC | None | None-Recall 5 years | No | No | 30 | No | No | Yes | No |
| 51 | M | Caucasian | Screening | 2015 | 10mm SSA/P Cecum, SSA/P-AC, 5mm SSA/P SC, 6mm TA-Rectum | None | None-Recall 3 years | No | No | 28 | Yes(mvt) | No | No | No |
| 52 | M | Caucasian | Surveillance | 2015 | 15mm SSA/P-Cecum | 2012: Diminutive-TA- TC | 2016: No polyps, 2017: 5mm TA-Rectosigmoid | No | No | 36 | No | No | Former | No |
| 35 | F | Caucasian | Abdominal pain | 2012 | 8mm HP-Cecum | None | None-Recall 10 years | No | No | 23 | No | No | Not availab | No |
| 64 | F | Caucasian | Surveillance | 2015 | 21mm SSA/P-HF | Yes-Not available | 2016-No polyps | Yes | No | 23 | No | No | No | No |
| 31 | F | Caucasian | Surveillance | 2014 | Multiple polyps | Yes-Multiple polyps | Yes-multiple polyps | No | No | 22 | No | No | No | Yes |
| 50 | F | Caucasian | Screening | 2015 | 6mm SSA/P-Cecum, 3mm TA- SC, 2mm TA-Rectum | None | None-Recall 3 years | No | No | 21 | No | No | No | No |
| 51 | M | Caucasian | Surveillance | 2015 | 5mm SSA/P-Cecum | 2011-Sigmoid  Colon cancer | 2016-4mmTA-DC | Yes | No | 26 | No | Colon cancer | No | No |
| 59 | M | Caucasian | Screening | 2016 | 6mm SSA/P Cecum | None | None-Recall 5 years | Yes | Yes | 31 | No | No | Yes | No |
| 64 | F | Caucasian | Surveillance | 2015 | 15mm SSA/P HF | 2014:SSA/P-10mm AC, SSA/P-14mm-HF | 2016:10mm SSA/P-HF, SSA/P-  4mm-DC | No | No | 28 | Yes(mvt) | No | No | No |
| 59 | F | Caucasian | Screening | 2016 | 25mm SSA/P-Cecum | None | 2017:8mm SSA/P cecum, 6mm TA-TC, 5mm TA-Rectum | No | No | 24 | No | No | Former | No |
| 72 | M | Caucasian | Surveillance | 2017 | 6mmSSA/P AC, Three 2-3mm-TA- TC, Multiple 2-3mm TA-Rectum | polyp at HP-DC, Two 3 to 10 mm HP- Rectum, Two 3-4mm | None-Recall 2 years | No | No | 19 | No | No | Yes | No |
