# Supplementary material for: Molecular Biomarkers of Sessile Serrated Adenoma/Polyps
Source: Clin Transl Gastroenterol. 2019 Nov 26;10(12):e00104. doi: 10.14309/ctg.0000000000000104 (PMC6970553; doi:10.14309/ctg.0000000000000104)
Supplement: SUPPLEMENTARY MATERIAL [file ct9-10-e00104-s010.docx]

Supplemental Figure 1:

Flow diagram that shows numbers of samples from different categories that were analyzed in this study, those used in the training and test sets, and which were analyzed for *BRAF* and *KRAS* mutations.

Supplemental Figure 2:

RT-qPCR results for each gene for all of this study’s 223 colon samples. They are shown in their sample categories that include the 3, 2, 1 or 0 –of-3-criteria classifications of all serrated polyps (training and test sets combined): 3 of 3 (n=49), 2 of 3 (n=38), 1 of 3 (n=18), 0 of 3 (n=72). Also shown are uninvolved (non-polyp, normal) tissues from patients with serrated polyps (n=27), and 19 “control” (non-polyp, normal), tissues from patients with no polyps found upon biopsy or known prior.

Supplemental Figure 3:

RT-qPCR results for each gene for all of this study’s 106 left-colon samples. They are shown in their four sample categories: L-SSA/P: small, left, SSA/P-morphology polyps (n=14); L-HP: small, left-colon HP-morphology polyps (n=72); L-Uninv: uninvolved (non-polyp, normal), left-colon tissues from patients with serrated polyps (n=11); “control” (non-polyp, normal), left-colon tissues from patients with no polyps found upon biopsy or known prior (n=9). Fold change and Mann-Whitney U-test p value are shown for each gene’s L-SSA/P / L-HP comparison. The three most HP-like L-SSA/P polyps and the six most SSA/P-like polyps according to ZIC2 relative level are shown as green and red dots, respectively, for all seven genes.

Supplemental Figure 4:

RT-qPCR results for each gene for his study’s 77 serrated polyps that were typed for *BRAF* V600E and *KRAS* Codons 12-13 mutations. Polyps are designated according to the 3, 2, 1 or 0 –of-3-criteria classifications. 3-of-3 and 2-of-3 –criteria polyps are combined here. The labels “mut” and “wt” denote mutant and wild-type, respectively. Mann-Whitney U-test p values of <0.05 for BRAF or KRAS –mutant polyps compared to wildtype, within the same 3-criteria class, are shown as asterisks above the mutant polyps’ group. (These results’ group sizes, mutant-vs.-wild-type fold changes, and p values are specified in Supplemental Table 4.)
